# Supplementary material for: The added value of right ventricular function normalized for afterload to improve risk stratification of patients with pulmonary arterial hypertension
Source: PLoS One. 2022 May 19;17(5):e0265059. doi: 10.1371/journal.pone.0265059 (PMC9119555; doi:10.1371/journal.pone.0265059)

S1 Fig: Kaplan-Meier curves according to risk re-stratification for TAPSE/sPAP for: (a) COMPERA registry, (b) invasive FPHN and (c) non-invasive FPHN registry


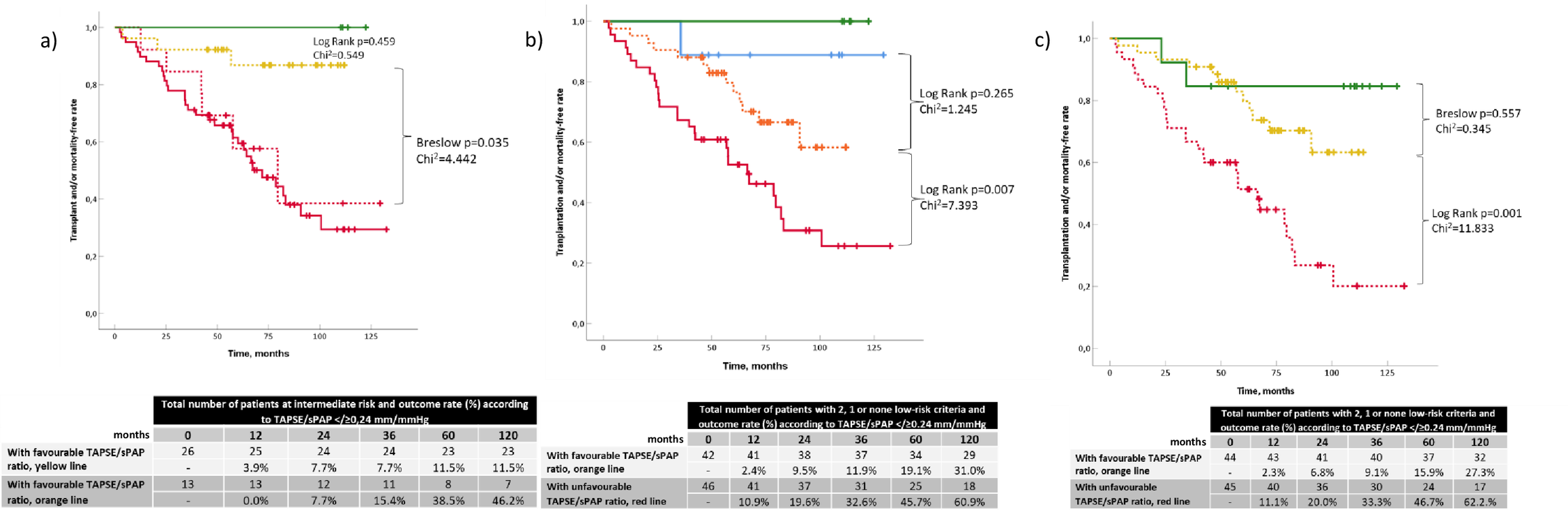

Supplement: S1 Fig — Kaplan-Meier curves according to risk re-stratification for TAPSE/sPAP for (a) COMPERA registry, (b) invasive FPHN and (c) non-invasive FPHN registry. (DOCX) [file pone.0265059.s001.docx]
