# Supplementary material for: The added value of right ventricular function normalized for afterload to improve risk stratification of patients with pulmonary arterial hypertension
Source: PLoS One. 2022 May 19;17(5):e0265059. doi: 10.1371/journal.pone.0265059 (PMC9119555; doi:10.1371/journal.pone.0265059)
Supplement: S3 Table — (DOCX) [file pone.0265059.s004.docx]

S3 Table: univariate regression analysis of hemodynamic data.

|  | HR | 95% CI | p |
| --- | --- | --- | --- |
| mPAP, mmHg | 1.010 | 0.992 - 1.027 | 0.276 |
| RAP, mmHg | 1.094 | 1.027 - 1.165 | 0.005 |
| PAWP, mmHg | 1.093 | 0.996 - 1.200 | 0.060 |
| CO, l/min | 0.793 | 0.623 - 1.009 | 0.059 |
| CI, l/min/m^2^ | 0.600 | 0.374 – 0.963 | 0.034 |
| PVR, UW | 1.043 | 0.988 - 1.102 | 0.125 |
| SvO_2_, % | 0.967 | 0.941 - 0.993 | 0.015 |
| SaO_2_, % | 0.934 | 0.875 - 0.998 | 0.042 |
| HR, bpm | 0.994 | 0.968 - 1.021 | 0.667 |
| SV, ml | 0.989 | 0.972 - 1.006 | 0.208 |
| SVI, ml/m^2^ | 0.970 | 0.937 - 1.005 | 0.091 |
| PP, mmHg | 1.010 | 0.989 - 1.032 | 0.343 |
| Ca, ml/mmHg | 0.362 | 0.170 - 0.775 | 0.009 |
| RC time, s | 0.337 | 0.052 - 2.289 | 0.255 |

The table shows the univariate regression analysis of the invasive data.

mPAP: mean pulmonary arterial pressure; RAP: right atrial pressure; PAWP: pulmonary arterial wedge pressure; CO: cardiac output; CI: cardiac index; PVR: pulmonary vascular resistance; SvO2: mixed venous oxygen saturation; SaO2: arterial oxygen saturation; HR: heart rate; SV: stroke volume; SVI: indexed stroke volume; PP: pulse pressure; Ca: pulmonary arterial compliance; RC time: resistance-compliance constant time.
